# Chemical Profile, Antibacterial Activity and Antibiotic-Modulating Effect of the Hexanic *Zea Mays* L. Silk Extract (Poaceae)

**DOI:** 10.3390/antibiotics8010022

**Published:** 2019-03-12

**Authors:** Ana Beatriz Linard de Carvalho, Cleciana Alves Cruz, Cicero Lucas Almeida de Freitas, José Junior dos Santos Aguiar, Paula Leticia Wendy de Souza Nunes, Valéria Maria da Silva Lima, Edinardo Fagner Ferreira Matias, Débora Feitosa Muniz, Henrique Douglas Melo Coutinho

**Affiliations:** 1 Department of Nursing, Universitary Center Dr. Leão Sampaio, Juazeiro do Norte 63040-005, Brazil; bia-linard@hotmail.com (A.B.L.d.C.); josejunior@leaosampaio.edu.br (J.J.d.S.A.); paulaleticiawendy@hotmail.com (P.L.W.d.S.N.); valeriasilvalima2016@outlook.com (V.M.d.S.L.); 2 Department of Nursing, Vale do Salgado College, Icó 63430-000, Brazil; clecianacruz@fvs.edu.br; 3 Department of Biomedicine, Universitary Center Dr. Leão Sampaio, Juazeiro do Norte 63040-005, Brazil; cicerolucasalmeida@yahoo.com.br (C.L.A.d.F.); effm_biologia@hotmail.com (E.F.F.M.); 4 Department of Biological Chemistry, Regional University of Cariri, Crato 63105-000, Brazil; deehmuniz78@gmail.com

**Keywords:** bacterial resistance, hexanic extract, *Zea mays* L.

## Abstract

The present study aimed to determine the chemical profile and to evaluate the antibacterial activity and antibiotic-modulating action of the hexanic *Zea mays* silk extract in association with aminoglycosides. Standard *Escherichia coli* ATCC 25922, *Staphylococcus aureus* ATCC 25923 and *Pseudomonas aeruginosa* ATCC 27853 strains, as well as multi-resistant *Escherichia coli* 27, *Staphylococcus aureus* 35, and *Pseudomonas aeruginosa* 31 strains, were used in this study. Phytochemical prospection revealed the presence of the following secondary metabolites: tannins, flavones, flavonoids, and xanthones, with the main chemical constituents being identified in plant extracts obtained with apolar organic solvents such as hexane. The extract presented a minimum inhibitory concentration (MIC) ≥1024 μg/mL against all the tested strains. The association of the extract with aminoglycoside antibiotics showed significant synergistic effects against *Staphylococcus aureus* and *Pseudomonas aeruginosa*, except for amikacin, which was antagonized by the extract against *E. coli.* These results indicate the *Zea mays* silk presents bioactive compounds with antibiotic-modulating properties. However, further research is required to characterize the effects of isolated compounds and determine their potential for drug development.

## 1. Introduction

Medicinal plants have been widely used for therapeutic purposes throughout generations [1] and for many people constitute an essential patrimony that must be preserved. In fact, the study of plant pharmacological properties has contributed significantly to the treatment of various diseases [2].

The *Zea mays* L. (POACEAE) species, popularly known as “corn” (“*milho*” in Portuguese) is widely used in Brazilian folk medicine to treat genitourinary tract diseases. This species is characterized as a monoecious plant which produces fruits and seeds [3,4]. Its stem is erect with an average height of 1.30 to 2.5 m and the leaves may be light green or dark green in color. *Zea mays* L. presents masculine flowers (arrows or pennants) at its highest part, where pollen grains are produced, and female flowers (spikes) along the stem. Each silk strand is responsible for grain production after fertilization. The silk is an elongated stylet that emerges from the tip of the spike from each female flower [4,5]. 

Corn silk is used by people from different countries to control blood pressure and treat a series of conditions, such as fever, gout and urinary tract infections, in addition to other bacterial diseases [6,7].

In studies performed with *Zea mays* silk extract against *Staphylococcus aureus*, *Escherichia coli* and *Pseudomonas aeruginosas* standard and multiresistant strains obtained significant results for the extract’s bacterial resistance modulatory activity, when tested alongside aminoglycoside antibiotics [8].

One of the greatest challenges in the treatment of bacterial infections is antibiotic resistance. While most microorganisms die when exposed to therapeutic doses of conventional antibiotics, some microorganisms become resistant to these drugs, in addition to transferring resistance mechanisms to their descendants [9]. Bacterial resistance is particularly important in opportunistic infections, such as those caused by *Staphylococcus aureus*. In this condition, microorganisms which are part of the normal microbiota can cause infectious diseases in immunosuppressed patients [10].

In this context, the study of natural products as an alternative to bacterial resistance has been obtaining promising results. In fact, medicinal plants present constituents capable of inhibiting or controlling bacterial resistance mechanisms, which can be obtained and tested at low cost [11].

Therefore, the objective of this study was to characterize the phytochemical profile and to evaluate the antibacterial and antibiotic-modulating activities of the hexanic *Zea mays* L. silk extract against standard and multidrug-resistant *Escherichia coli*, *Staphylococcus aureus* and *Pseudomonas aeruginosa* strains.

## 2. Results 

Phytochemical prospection of the hexanic *Zea mays* silk extract (HEZM) revealed the presence of four important secondary metabolites, including: tannins, flavones, flavonols, and xanthones (Table 1).

The microdilution test was used to determine the minimum inhibitory concentration (MIC) of the HEZM against standard and multiresistant *E. coli*, *S. aureus* and *P. aeruginosa* strains obtaining MIC values ≥1024 for all tested strains. As shown in Table 2, the extract presented MIC values ≥ 1024 μg/mL against all evaluated strains, indicating that at least for these microorganisms, the extract does not present a significant antibacterial effect. 

**Figure 1:**
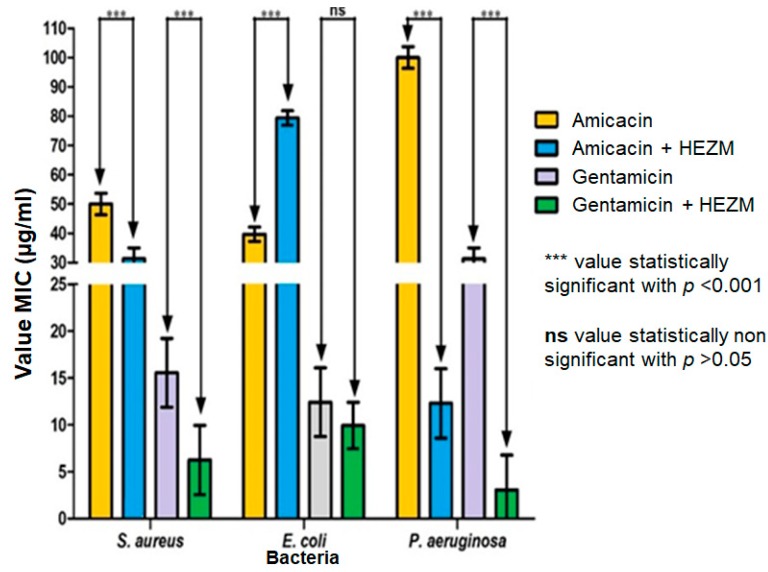
Antibiotic-modulating effect of HEZM against multiresistant bacteria.

To investigate the HEZM modulatory effect, a subinhibitory concentration (MIC/8) of this extract was used in combination with the amikacin and gentamicin antibiotics against multiresistant bacterial strains (Figure 1). The results showed that the association of the extract with antibiotics significantly decreased the MIC of the antibiotics against *Staphylococcus aureus* and *Pseudomonas aeruginosa* strains, indicating the antibiotics had their effects potentiated by the extract. However, the association of the extract with amikacin against *Escherichia coli* significantly increased the MIC of this antibiotic, indicating the extract antagonized the effect of the drug. Together, these results indicate that although it does not exhibit a significant antibacterial effect, the extract is capable of selectively modulating bacterial resistance against aminoglycosides (Figure 1).

## 3. Discussion

According to [12], saponins, calcium, sodium, magnesium, and proteins can also be found in this extract. The flavonoids found in this study belong to an important natural metabolite group common in plant leaves, flowers and roots with effective modulatory potential capable of inhibiting many cellular system enzymes [13]. Xanthones, another metabolite present in the extract, has a family subgroup known as oxygenated xanthones that present excellent antimicrobial action against *S. aureus* strains resistant to norfloxacin, mainly because this type of metabolite presents a hydrophobic property that increases cellular membrane solubility [14]. Tannins found in the present extract have the ability to form protein complexes capable of controlling fungi and bacteria [15,16]. Nishizawa (1990) reports that a number of bacteria are sensitive to tannins at minimum concentrations (0.5 g/L), including *Staphylococcus aureus*, *Streptococcus pneumonia*, *Bacillus anthracis*, and *Shigella dysenteriae*. 

The synergistic effect obtained from the association of the extract with antibiotics in this study may be due to the action of metabolites present in the extract, such as tannins. According to [17], tannins can precipitate proteins and interfere with cellular components in microorganisms, including *Staphylococcus aureus*, *Streptococcus pneumoneae*, and *Shigella dysenteriae*. In addition, the extract contains flavonoids, whose antimicrobial properties have been previously described [18,19].

The antimicrobial properties present in tannins appear to be associated with the hydrolysis of an ester link in gallic acid, which confers natural defense properties against microbial infections to plant products. Meanwhile, metabolites such as flavonoids, which act at the plasmatic wall of microorganisms, due to their lipophilic affinity, tend to be synthesized in response to infections [20,21].

The association of extracts with aminoglycosides represents a promising therapeutic option for infections caused by resistant bacteria since the potentiation of antibiotic effects can reduce the therapeutic dose and consequently, minimize their side effects [17].

On the other hand, the modulating effect of the extract appears to be different between Gram-positive and Gram-negative microorganisms, given the results with *Staphylococcus aureus* (Gram-positive) were different from the results with *E. coli* and *P. aeruginosa* (Gram-negative). In fact, Gram-negative bacteria present a thin peptidoglycan layer, whereas Gram-positive bacteria present a more complex lipid layer. Thus, these differences in the chemical components of these microorganisms may be the determinant factor in the antibiotic response [19].

These factors may also justify the antagonistic effect observed in the assays with *E. coli*, since the compounds present in the extract could interfere with the binding of amikacin to bacterial lipid compounds, decreasing the activity of this drug [22]. In addition, the tannins present in the extract could also interfere with the activity of the drug through a chelating effect [22].

## 4. Materials and Methods

### 4.1. Botanical Material

Corn silk samples were collected from a family farm located in a place known as “Sítio palmeiras” in the municipality of Crato-CE, Brazil. The geographic coordinates are Latitude: 7°13′46″ South and Longitude: 39°24′32″ West. A *sample specimen* was deposited in the “Dárdano de Andrade Lima” Herbarium, at the Regional University of Cariri—URCA under sample number: 13.351.

### 4.2. Hexanic Extract and Solution Preparations for Antibacterial Assays

For the preparation of the hexanic extract, 356 g of *Zea mays* silk were crushed and placed in a glass flask containing 1 L of hexane. The material was kept submerged in the solvent for at least 72 h and was then transferred to a rotary evaporator in a water bath to remove any organic solvent residue, yielding 12.46 g of a raw hexane extract, which corresponded to 3.5% of the total collected and processed silk. This extract was prepared to a concentration of 10 mg/mL, dissolved in dimethyl sulfoxide (DMSO) and diluted with distilled water to a concentration of 1024 μg/mL [23].

### 4.3. Extract and Phytochemical Prospection Acquisition

The hexane extract was obtained using *Zea mays* silk strands, for which the silk strands were collected, weighed and ground to increase their surface area, followed by deposition in a glass flask containing the organic solvent at a volume sufficient to submerge all the material. This was maintained closed for 72 h. Thereafter, the material was filtered using filter paper to eliminate possible solid residues with the contents later being concentrated in a vacuum rotary condenser (model Q-344B, Quimis, Campinas Brazil) and water bath (model Q-214M2, Quimis, Brazil). Phytochemical prospection was carried out according to the methodology proposed by [24] to investigate the presence of chemical compounds, such as coumarins, quinones, triterpenes, steroids, organic acids, and alkaloids. The tests are based on colorimetric changes or precipitate formation after the addition of specific reagents and pH variations, which can be visually evaluated.

### 4.4. Minimum Inhibitory Concentration (Mic) Determination

The minimum inhibitory concentration (MIC) of the hexanic *Zea mays* silk extract (HEZM) was determined through the broth microdilution assay [25,26,27]. A 100 μL volume of the inoculum from each strain was prepared in 10% BHI broth at a concentration of 10^5^ CFU/mL, placed in 96-well microdilution plates and serially diluted 1:2 [28]. The HEZM (100 mL) was added to the first well and diluted to reach final concentrations ranging from 512 μg/mL to 8 μg/mL. The last wells were reserved for the controls using the standard antibiotics amikacin and gentamicin, whose concentrations varied from 2500 μg/mL to 2.4 μg/mL. The plates were incubated at 37 °C for 24 h and after this period the results were evaluated by using resazurin as a colorimetric indicator of bacterial growth [29,30]. The readings were performed using a spectrophotometer and the MIC was defined as the lowest concentration capable of inhibiting bacterial growth [27]. Pilot tests were performed to evaluate the interference of DMSO, where at a concentration lower than 10% the solvent has no influence on the results. These tests were performed in triplicates using only multiresistant bacterial strains.

### 4.5. Aminoglycoside Antibiotic Resistance Modulation Analysis 

To analyze the HEZM effect on bacterial resistance to aminoglycoside antibiotics, the method proposed by [23] was used, where the HEZM was tested at a subinhibitory concentration (MIC/8). In addition to the extract solution, 100 μL of the bacterial inoculum solution in 10% BHI were added to the wells. Antibiotic solutions were prepared using sterile distilled water at a concentration of 5000 μg/mL. Then, 100 μL of this solution was added to the first well and serially diluted (1:2) up until the penultimate well to reach concentrations ranging from 2500 to 2.44 μg/mL. The last well was reserved for the control [23], where these were expected to grow in the absence of antibiotics, thus validating the tests. The plates were incubated at 37 °C for 24 h and readings were performed as described above. These tests were performed in triplicates using only multiresistant bacterial strains (Table 3).

### 4.6. Statistical Analysis

The results from the assays were expressed as the geometric mean of triplicates. The data were analyzed using an ANOVA followed by Bonferroni’s post-hoc test using the GraphPad Prism 6.0 software. Differences with a *p* < 0.05 were considered significant.

### 4.7. Data Statement

The quantitative data used to support the findings of this study are included in the article.

## 5. Conclusions

In conclusion, the hexanic *Zea mays* L. silk extract did not present a clinically significant antibacterial effect against standard and multiresistant bacteria. However, the extract selectively modulated the action of aminoglycoside antibiotics against multiresistant bacteria, indicating that its components present antibiotic-modulating effects.

Therefore, further research is required to isolate the extract’s bioactive compounds and identify which constituents promote synergism or antagonism in association with antibiotics. Thus, chemical components of the *Zea mays* silk extract may be used in the development of novel drugs useful for the treatment of infections caused by microorganisms resistant to conventional antibiotics.

## Figures and Tables

**Table 1:** Phytochemical prospection of the hexanic *Zea mays* L. extract.

Extract	1	2	3	4	5	6	7	8	9	10	11	12
Hezm	-	+	-	-	+	+	+	-	-	-	-	-

1-Phenols; 2-Pyrogallic Tannins; 3-Anthocyanins; 4-Anthocyanidinis; 5-Flavones; 6-Flavonols; 7-Xanthones; 8-Chalcones; 9-Aurones; 10-leucoanthocyanidins; 11-Catechins; 12-Alkaloid; (+) Presence; (-) Absence. HEZM-Hexanic Extract of *Zea mays* L.

**Table 2:** The hexanic *Zea mays* silk extract (HEZM) minimum inhibitory concentration (MIC) against standard and resistant bacterial strains.

Bacterium	MIC (μg/mL)
*Escherichia coli* ATCC 25922	≥1024
*Escherichia coli* 27	≥1024
*Staphylococcus aureus* ATCC 25923	≥1024
*Staphylococcus aureus* 35	≥1024
*Pseudomonas aeruginosa* 31	≥1024
*Pseudomonas aeruginosa* ATCC 27853	≥1024

**Table 3:** Resistance profile of the bacteria used in the tests.

Bacterium	N	Collection Site	Resistance Profile
*Staphylococcus aureus*	SA358	Surgical wound	Oxa, Gen, Tob, Ami, Can, Neo, Para, But, Sis, Net
*Pseudomonas aeruginosa*	P31	Nose	Pol, Cpm, Ctz, Ptz, Ami Imi, Cip, Lev, Mer,
*Escherichia coli*	EC27	Surgical wound	Ast, Ax, Amp, Ami, Amox, Ca, Cfc, Cf, Caz, Cip, Clo, Imi, Can, Szt, Tet, Tob

Resistance Profile: Ami = amikacin; Sam = ampicillin-sulbactam; Cip = ciprofloxacin; Lev = levofloxacin; Cpm = cefepime; Ctz = ceftazidime; Pol = polymyxin; Imi = imipenem; Mer = meropenem; Ptz = piperacillin; Tig = tigecycline; Ast = aztreonan; Ax = Amoxicillin; Amp = ampicillin; Amox = amoxilin, Ca = cefadroxil; Cfc = cefaclor; Cf = cephalothin; Caz = ceftazinidime; Clo = chloramphenicol; Can = kanamycin; Szt = sulfametrim; Tet = tetracycline; Tob = tobramycin; Oxa = oxacillin; Gen = gentamicin; Neo = neomycin; Para = paramomycin; But = butyrosine; Sis = sisomycin; Net = netilmicin. The microorganisms used in this research were acquired from the Laboratory of Mycology of the Federal University of Paraíba—UFPB and kindly provided by the Regional University of Cariri—URCA.
